# The Swedish version of the multidimensional scale of perceived social support (MSPSS) - a psychometric evaluation study in women with hirsutism and nursing students

**DOI:** 10.1186/1477-7525-11-168

**Published:** 2013-10-10

**Authors:** Maria Ekbäck, Eva Benzein, Magnus Lindberg, Kristofer Årestedt

**Affiliations:** 1 Department of Dermatology, University Hospital Örebro and School of Health and Medical Sciences, Örebro University, Örebro, SE 70185, Sweden; 2 School of Health and Caring Sciences, Linnaeus University, Kalmar, Sweden; 3 Department of Medical and Health Sciences, Division of Nursing Science, LinköpingUniversity, Linköping, Sweden; 4 Palliative Research Centre, Ersta Sköndal University College and Ersta hospital, Stockholm, Sweden

**Keywords:** Psychometrics, Validation, Hirsutism, Social support, Translations

## Abstract

**Background:**

The Multidimensional Scale of Perceived Social Support (MSPSS) is a short instrument, developed to assess perceived social support. The original English version has been widely used. The original scale has demonstrated satisfactory psychometric properties in different settings, but no validated Swedish version has been available. The aim was therefore to translate, adapt and psychometrically evaluate the Multidimensional Scale of Perceived Social Support for use in a Swedish context.

**Method:**

In total 281 participants accepted to join the study, a main sample of 127 women with hirsutism and a reference sample of 154 nursing students. The MSPSS was translated and culturally adapted according to the rigorous official process approved by WHO. The psychometric evaluation included item analysis, evaluation of factor structure, known-group validity, internal consistency and reproducibility.

**Results:**

The original three-factor structure was reproduced in the main sample of women with hirsutism. An equivalent factor structure was demonstrated in a cross-validation, based on the reference sample of nursing students. Known-group validity was supported and internal consistency was good for all scales (α = 0.91-0.95). The test-retest showed acceptable to very good reproducibility for the items (κ_w_ = 0.58-0.85) and the s**c**ales (ICC = 0.89-0.92; CCC = 0.89-0.92).

**Conclusion:**

The Swedish version of the MSPSS is a multidimensional scale with sound psychometric properties in the present study sample. The simple and short format makes it a useful tool for measuring perceived social support.

## Background

Each individual interacts in different social relationships, for example with family and friends. When living with a life-long medical disease, the perceived support from these social relationships is of the utmost importance, as several studies have documented that social support has a great impact on the physical health outcome [1-3]. In addition, there is evidence that social support has effects on the cardiovascular response [4] and also on the neuroendocrine and immune functions [5]. It is further argued that social support has a buffering effect on stressful life events and depression [6] and that perceived social support promotes self-esteem, which in turn has a positive effect on mental health [7].

Social support is a multidimensional concept with different definitions and ways of measuring it [8], although there is a consensus among researchers that social support involves some kind of relationship transaction between individuals [9,10]. There are different types of supportive functions provided through social relationships. Tardy [11] suggested that social support can be divided into five key dimensions: direction of social support (given or received), disposition (availability vs. utilization), description of the support vs. the satisfaction with the utilized support, content of support (type or kind of support), and network (in what social system the support is provided). Both quantitative and qualitative measures of social support have been investigated [12]. However the perceived social support seems to be the most important for the individual receiving the support [13].

The perceived social support has different functions, i.e. emotional support, instrumental support (practical support), informative support and appraisal support [8]. Individuals participate in different social networks during working time and spare time and different forms of support are given. Partners and close family seem to give the greatest contribution in chronic disease. In networks with no partners other people seem to contribute more in illness-related work [14]. However, there seem to be cultural differences. For example, young female Asian adults perceived the highest social support from friends [15], while young Arabian Americans perceived highest support from their family [16]. For a better understanding of health, social support should be taken into consideration by both researchers and clinicians. Assessment of perceived social support can be one way to do this.

There are several instruments that aim to measure social support. A promising scale that has been widely used for some decades is the Multidimensional Scale of Perceived Social Support (MSPSS) constructed by Zimet et al., in 1988 [9]. In relation to different sources of support (family, friends and significant others, the MSPSS assesses both perceived availability and adequacy of emotional and instrumental support. This instrument is preferably short and has been found to be reliable and valid both in its original language [17] and in other language versions [18,19]. The instrument has been used in clinical and non-clinical settings [3,20,21], in various age groups [7,22,23] and in samples with various cultural backgrounds [24-26]. Until today, there is no validated Swedish version of the MSPSS, a prerequisite for international communication and comparisons between studies. The aim of this study was therefore to translate, adapt and psychometrically evaluate the Multidimensional Scale of Perceived Social Support for use in a Swedish context.

## Methods

### Measures

#### The multidimensional scale of perceived social support (MSPSS)

The MSPSS was developed by Zimet, Dalhem, Zimet, & Farley (1988) [9] and aims to measure perceived social support. It includes 12 items which cover three dimensions; Family, Friends and Significant others. Each item is rated on a seven-point Likert-type response format (1 = very strongly disagree; 7 = very strongly agree). A total score is calculated by summing the results for all items. The possible score range is between 12 and 84, the higher the score the higher the perceived social support. In addition, separate subscales can be used by summing the responses from the items in each of the three dimensions. The possible score range for the subscales/dimensions is between 4 and 28 [9,17]. The MSPSS is widely used and the three-factor model has demonstrated good psychometric properties in previous studies. There is less evidence available for the one-factor model [9,16,18,23].

#### Translation

Translation and cultural adaptation of the MSPSS was carried out according to WHO: s official process of translation and adaptation of research instruments [27]. Four researchers, two physicians and two nurses individually translated the instrument from English to Swedish. These versions were discussed in the research group and a decision on an agreed upon version was made. A bilingual person, familiar with both cultures and a native English speaker, compared the Swedish agreed-upon version and the English original version. An independent authorized translator then performed a back translation. The two versions were compared and there was a slight difference regarding the translation of the items in the dimension “Significant others” (items 1, 2, 5, & 10). In those items, “a special person” was back translated as “an important person in my life close by”. This difference was discussed with the constructor, Professor Zimet, who claimed that although those four items deviate slightly from the original wording, the item still had the same meaning and reflected the underlying construct, i.e. perceived social support from an important person in one’s life. This version has then been pretested in a group of 30 Swedish nursing students in order to evaluate the clarity of instructions, items and response format.

### Sample and procedure

Two different samples were included to evaluate the psychometric properties of the MSPSS. The main sample was taken from an ongoing observational study of health-related quality of life among women with hirsutism. The sample was recruited between October 2010 and February 2012*.* Inclusion criteria were being diagnosed with hirsutism in the medical records at a Dermatology Department, or having on-going laser treatment for excessive hair growth at a private laser clinic. In total 200 identified women received an invitation to participate. Exclusion criteria were not having the diagnosis hirsutism (misdiagnosed - which was shown in the questionnaire), severe psychiatric disease, or inability to speak and understand Swedish. Hair growth was self-reported according to the Ferriman-Gallwey score (FG), which is a well-established visual scoring method, developed by Ferriman and Gallwey and further modified by Hatch and Tredway [28]. Nine body areas are scored from 0 to 4 and the total possible score is 36. Hirsutism was defined as a FG ≥ 6 or FG ≥ 2 in facial hirsutism. Of the 200 invited women, 132 (66%) returned the acceptance form. Because of the above mentioned exclusion criteria, five women were excluded. The sample finally consisted of 127 women (65%). The included settings were two dermatological departments at university hospitals and one private dermatological laser clinic, of which two were situated in the middle of Sweden and one in the southern region. The study was approved by the Regional Ethical Review Board at Uppsala University (study code 2010/207).

A sample of nursing students was added as a reference group and to make it possible to cross-validate the MSPSS scores in another sample. Nursing students were recruited between November 2010 and December 2011. Included students were in the middle of a three year nursing programme. Students from three classes were asked to participate (n = 168), of which 154 (92%) accepted participation. Students in one of the three classes were also asked to respond to the questionnaire once again one week after the first occasion. Of the 58 students who responded to the first questionnaire, 44 also completed the questionnaire after seven days. In addition to the MSPSS, the questionnaire also included questions about age, gender, having a partner and having children.

### Statistical analyses

The psychometric evaluation of the MSPSS started with an item analysis on the main sample of women with hirsutism. This analysis included a description of the score distribution for items and scales. The Shapiro-Wilk W test was conducted to evaluate if the scale scores deviated from a normal distribution [29]. The associations between items were evaluated with inter-item correlations while the associations between items and scales were evaluated with item-total correlations corrected for overlap. Both tests are based on Pearson’s product–moment correlations. An acceptable level of the item-total correlations was set to r > 0.3 [30].

An exploratory factor analysis, based on a principal-component factor method, was conducted to evaluate the factor structure of the MSPSS [30,31]. An orthogonal varimax rotation was conducted to facilitate the interpretation of the factor structure [32]. The factor analysis was first conducted on the main sample to evaluate if the original factor structure could be reproduced. Thereafter, the factor analysis was cross-validated in the reference sample to evaluate if the factor structure was stable during other conditions, i.e. in the nursing students. Data for the women with hirsutism and nursing students respectively were first examined with Bartlett’s test of sphericity (χ^2^ (66) =1662.1, p < 0.001 vs. χ^2^ (66) =1711.4, p < 0.001) and with Kaiser-Meyer Olkin measure (KMO) in each item (0.86-0.93 vs. 0.80-0.90) and all items together (0.89 vs. 0.87). All these examinations indicated great sampling adequacy [32]. The number of factors extracted was decided by the Kaiser criteria (eigenvalue >1.0) and controlled with Horn’s parallel analysis [33]. The parallel analyses were based on 1000 iterations, using the mean estimate. For the main sample, this test showed a clear cutoff at three factors with adjusted eigenvalues between 1.062 and 6.760. The adjusted eigenvalue for the fourth factor was 0.165.

Student’s t-test and Cohen’s d effect size were used to evaluate if the MSPSS was able to detect differences between groups [34] i.e., known group validity. As the assumption of homogeneity of variance was violated, all t-tests were corrected using Satterthwaite’s degree of freedom [35]. Based on previous studies [36,37], we hypothesized that women with hirsutism should score significant, and clinically relevant, lower social support compared to the nursing students. The interpretation of Cohen’s d effect size was as following; low (0.2) medium (0.5) or high (0.8) [38].

Reliability was evaluated according to internal consistency and intra-rater reliability (test-retest reliability). Internal consistency was evaluated using Cronbach’s alpha and a 95% confidence interval. The confidence interval was calculated using bootstrapping with 1.000 replications. Intra-rater reliability was evaluated for both item responses and scale scores. According to their ordinal level, the items were evaluated by weighted kappa statistics (κ_w_), using linear weights for agreement [39]. The scale scores were evaluated with intraclass correlations for paired measurements (ICC, one-way model) and Lin’s concordance coefficients (CCC). A 95% confidence interval was calculated for both κ_w_, ICC and CCC. To indicate adequate reproducibility for items, we used the following interpretation of the κ_w_ statistics: poor (0.00-0.20), fair (0.21-0.40), moderate (0.41-0.60), good (0.61-0.80) and very good (0.81-1.00) [40]. For scales, ICC and CCC should be beyond 0.7 to indicate satisfactory reproducibility [41].

All statistical analyses were conducted with Stata 12.1 (StataCorp LP, College Station, TX, USA). Horn’s parallel analysis was conducted with PARAN, an additional command for Stata [33]. The level of statistical significance was overall set at p < 0.05.

## Results

### Characteristics of the sample

Totally 281 persons were included in the study, a main sample of 127 women with hirsutism and a reference sample of 154 nursing students. The women with hirsutism were significantly older compared to the nursing students, median age 32.0 and 23.5 respectively. Having children were also significantly more common among the women with hirsutism compared to the nursing students. There was no significant difference between the groups for living with a partner, 45% and 51% respectively (Table 1).

**Table 1:** Characteristics of the participants in the main and reference sample

	**Women with hirsutism**	**Nursing students**	**p-value**
	**(n = 127)**	**(n = 154)**	
Age, median (q1-q3) [mean ± SD]	30 (24–39) [32.0 ± 10.3]	23.5 (22–31) [27.3 ± 7.8]	<0.001^a^
Sex, n (%)			
Women	127 (100.0)	136 (88.3)	N/A
Man		18 (11.7)	
Living with a partner, n (%)			0.336^b^
Yes	57 (44.9)	78 (50.6)	
No	70 (55.1)	76 (49.4)	
Having children, n (%)			<0.001^b^
Yes	57 (44.9)	46 (29.9)	
No	70 (55.1)	108 (70.1)	
FG score, median (q1-q3) [mean ± SD]	18 (12–25) [18.8 ± 8.4]	-	N/A
FG score = Ferriman-Gallwey score			
N/A = not applicable			
q1-q3 = quartile I and III			

^a^Mann–Whitney U test.

^b^χ^2^-test.

### Item analysis

The findings from the item analysis on the main sample of women with hirsutism are present in Tables 2 &3. The items with lowest mean scores all belonged to the MSPSS Friends subscale while the highest mean scores were shown in items belonging to the MSPSS Significant others subscale (Table 2).

**Table 2:** Descriptive statistics and item-total correlations based on the main sample (women with hirsutism, n = 127)

					**Score distibutaion, n (%)**		
**Items and scales**		**Subscales**	**Mean (SD)**	**ITC**^**a**^	**Lowest**	**Highest**	**Missing**
1	There is a special person who is around when I am in need	SO	5.73 (1.78)	0.749	7 (5.5)	70 (55.1)	-
2	There is a special person with whom I can share my joys and sorrows	SO	5.68 (1.83)	0.759	8 (6.3)	67 (52.8)	-
3	My family really tries to help me	Fam	5.64 (1.74)	0.734	5 (3.9)	65 (51.2)	-
4	I get the emotional help and support I need from my family	Fam	5.23 (1.92)	0.773	7 (5.5)	53 (41.7)	-
5	I have a special person who is a real source of comfort to me	SO	5.62 (1.74)	0.775	5 (3.9)	62 (48.8)	-
6	My friends really try to help me	Fri	4.49 (2.03)	0.753	15 (11.8)	31 (24.4)	-
7	I can count on my friends when things go wrong	Fri	4.69 (2.21)	0.700	20 (15.8)	41 (32.3)	1 (0.8)
8	I can talk about my problems with my family	Fam	5.08 (2.07)	0.738	10 (7.9)	53 (41.8)	1 (0.8)
9	I have friends with whom I can share my joys and sorrows	Fri	4.79 (2.13)	0.723	15 (11.8)	43 (33.9)	2 (1.6)
10	There is a special person in my life who cares about my feelings	SO	5.69 (1.75)	0.739	4 (3.2)	68 (53.5)	1 (0.8)
11	My family is willing to help me make decisions	Fam	5.34 (1.92)	0.647	8 (6.3)	59 (46.5)	1 (0.8)
12	I can talk about my problems with my friends	Fri	4.51 (2.19)	0.721	19 (15.0)	37 (29.1)	1 (0.8)
	MSPSS Total (possible score range 12–84)		62.50 (18.26)				
	MSPSS Family (possible score range 4–28)		21.22 (7.03)				
	MSPSS Friends (possible score range 4–28)		18.50 (7.99)				
	MSPSS Significant Others (possible score range 4–28)		22.72 (6.66)				

Fam = Family; Fri = Friends; SO = Significant others.

^a^Item-total correlations (corrected for overlaps) for the relation between each item and the total scale score.

**Table 3:** Inter-item correlations based on the main sample (women with hirsutism, listwise deletion, n = 123)

**Items**	**Item 1**	**Item 2**	**Item 3**	**Item 4**	**Item 5**	**Item 6**	**Item 7**	**Item 8**	**Item 9**	**Item 10**	**Item 11**	**Item 12**
Item 1	1.000											
Item 2	0.960	1.000										
Item 3	0.588	0.592	1.000									
Item 4	0.663	0.632	0.853	1.000								
Item 5	0.832	0.819	0.609	0.514	1.000							
Item 6	0.469	0.478	0.453	0.502	0.540	1.000						
Item 7	0.421	0.435	0.396	0.428	0.460	0.869	1.000					
Item 8	0.559	0.609	0.754	0.765	0.582	0.495	0.427	1.000				
Item 9	0.438	0.471	0.415	0.445	0.433	0.812	0.863	0.471	1.000			
Item 10	0.827	0.834	0.521	0.536	0.908	0.526	0.459	0.522	0.471	1.000		
Item 11	0.432	0.417	0.775	0.775	0.464	0.417	0.386	0.756	0.408	0.370	1.000	
Item 12	0.440	0.464	0.416	0.455	0.471	0.792	0.792	0.471	0.873	0.494	0.440	1.000

All correlation coefficients were significant at a level of p < 0.001.

Reflected by the mean values and the frequency of endorsement, all item scores were negatively skewed distributed with a ceiling effect. However, all response categories were used. Also the scale scores were negative skewed distributed, with a significant deviation from a normal distribution (p < 0.01). The number of missing data in the MSPSS was low (n = 7, <0.5%). All missing data were found in items 7–12, and related to four participants (1–3 missings each) (Table 2).

The associations between items were satisfactorily high. The inter-item correlation analysis showed that all 12 items correlated significantly (r = 0.37-0.96, p < 0.001) with one another. The strongest pairwise correlations were identified between items belonging to the same MSPSS subscale; Family 0.75-0.85, Friends 0.79-0.87, and Significant others 0.83-0.96. The pairwise correlations between items belonging to different subscales ranged from 0.37 to 0.67 (Table 3). The item-total correlations exceed the critical value of 0.3 for all items and range between 0.65 and 0.78 (Table 2).

### Factor structure

The original factor structure with three dimensions was reproduced in both the main sample of women with hirsutism and in the cross-validation, based on the reference sample of nursing students. In both groups, the three factor structure was supported by Horn’s parallel analysis. All items demonstrated factor loadings >0.7 on the dimensions they were expected to be related with, according to the original measurement model. No item demonstrated a factor loading ≥0.45 with a dimension it should not be related with. In the main sample of women with hirsutism, the strongest factor correlations were demonstrated between the subscales Friends and Significant others. In contrast, the strongest association was demonstrated between the subscales Family and Friends in the sample of nursing students (Table 4).

**Table 4:** Factor structure and internal consistency of the MSPSS in the main and reference sample

		**Women with hirsutism (n = 123)**				**Nursing students (n = 154)**			
		**Fam**	**Fri**	**So**	**Unique-ness**	**Fam**	**Fri**	**So**	**Unique-ness**
MSPSS Family (Fam)									
3	My family really tries to help me	0.833	0.185	0.361	0.142	0.891	0.134	0.261	0.121
4	I get the emotional help and support I need from my family	0.808	0.221	0.410	0.131	0.893	0.117	0.260	0.123
8	I can talk about my problems with my family	0.782	0.263	0.341	0.204	0.827	0.178	0.316	0.184
11	My family is willing to help me make decisions	0.894	0.230	0.129	0.132	0.768	0.193	0.302	0.281
MSPSS Friends (Fri)									
6	My friends really try to help me	0.226	0.856	0.278	0.139	0.218	0.814	0.328	0.182
7	I can count on my friends when things go wrong	0.169	0.905	0.208	0.109	0.103	0.899	0.208	0.137
9	I have friends with whom I can share my joys and sorrows	0.200	0.905	0.208	0.098	0.117	0.909	0.119	0.146
12	I can talk about my problems with my friends	0.219	0.868	0.227	0.147	0.137	0.919	0.133	0.120
MSPSS Significant others (So)									
1	There is a special person who is around when I am in need	0.299	0.201	0.872	0.111	0.376	0.181	0.815	0.162
2	There is a special person with whom I can share my joys and sorrows	0.295	0.229	0.864	0.115	0.381	0.161	0.767	0.241
5	I have a special person who is a real source of comfort to me	0.390	0.245	0.857	0.120	0.314	0.266	0.792	0.204
10	There is a special person in my life who cares about my feelings	0.174	0.283	0.891	0.096	0.247	0.306	0.797	0.210
	Explained variance after rotation	0.269	0.297	0.307	0.873^a^	0.283	0.289	0.252	0.824^a^
	Internal consistency								
	Cronbach’s alpha	0.93	0.95	0.95	0.94	0.92	0.94	0.91	0.92
	Cronbach’s alpha, 95% CI	0.90–0.95	0.93–0.96	0.93–0.97	0.92–0.96	0.89–0.95	0.91–0.95	0.87-0.95	0.89–0.94
	Factor correlations								
	Family	1.000				1.000			
	Friends	0.590	1.000			0.830	1.000		
	Significant others	0.558	0.867	1.000		0.552	0.590	1.000	

Uniqueness = 1-communality value.

^a^Total explained variance.

### Known group validity

The MSPSS had the ability to discriminate between women with hirsutism and nursing students. As hypothesized to support known group validity, women with hirsutism scored significantly lower social support compared to the nursing students in all MSPSS scales. According to the Cohen’s d effect size, all scales demonstrated clinically relevant differences between the main and reference sample. The greatest differences were demonstrated for the MSPSS Friends (d = 0.90, large effect) and smallest for MSPSS Family (d = 0.54, medium effect) (Table 5).

**Table 5:** Known group validity

**Scales**	**Women with hirsutism Mean (SD)**	**Nursing students Mean (SD)**	**t-value (df)**^**a**^	**p-value**	**ES**^**b**^
MSPSS Total	62.50 (18.25)	73.24 (10.59)	5.79 (185.79)	<0.001	0.85
MSPSS Family	21.22 (7.03)	24.12 (4.77)	3.92 (210.28)	<0.001	0.54
MSPSS Friends	18.50 (7.99)	23.44 (4.54)	6.11 (183.33)	<0.001	0.90
MSPSS Significant Others	22.72 (6.66)	25.69 (3.61)	4.49 (183.78)	<0.001	0.66

^a^All t-test were corrected for unequal variance using the Satterthwaite’s degree of freedom.

^b^Cohen’s d effect size (>0.5 medium effect; >0.8 large effect).

### Reliability

The reliability was satisfactory high for the MSPSS in the reference sample of nursing students (Tables 4 & 6). The internal consistency was high for all scales in both the main sample of women with hirsutism and the reference sample of nursing students. The internal consistency was higher in the main sample compared to the reference sample, but no MSPSS scale demonstrated a Cronbach’s alpha value <0.9 (Table 4).

The test-retest showed that MSPSS produced item and scale scores with satisfactory reproducibility. Except for item 5 (κ_w_ = 0.58), all others demonstrated good (κ_w_ = 0.6-0.8) or very good (κ_w_ = 0.8-1.0) reproducibility. Also the MSPSS scales demonstrated good reproducibility according to the intraclass and concordance correlation coefficients, which both demonstrated the same level of reliability. Both ICC and CCC ranged between 0.89 and 0.92 for the different MSPSS scales (Table 6).

**Table 6:** Test-retest reliability coefficients for the MSPSS items and scales based on the reference sample (nursing students, n = 44)

**Items and scales**		**κ **_**w **_**(95% CI)**^**a**^	**ICC (95% CI)**^**b**^	**CCC (95% CI)**^**c**^
1	There is a special person who is around when I am in need	0.74 (0.40–0.95)		
2	There is a special person with whom I can share my joys and sorrows	0.71 (0.54–0.90)		
3	My family really tries to help me	0.66 (0.47–0.80)		
4	I get the emotional help and support I need from my family	0.72 (0.59–0.85)		
5	I have a special person who is a real source of comfort to me	0.58 (0.34–0.74)		
6	My friends really try to help me	0.74 (0.61–0.87)		
7	I can count on my friends when things go wrong	0.68 (0.52–0.82)		
8	I can talk about my problems with my family	0.71 (0.54–0.84)		
9	I have friends with whom I can share my joys and sorrows	0.85 (0.75–0.94)		
10	There is a special person in my life who cares about my feelings	0.75 (0.49–0.94)		
11	My family is willing to help me make decisions	0.60 (0.40–0.80)		
12	I can talk about my problems with my friends	0.77 (0.66–0.88)		
	MSPSS Total		0.92 (0.86–0.96)	0.92 (0.88–0.97)
	MSPSS Family		0.92 (0.86–0.96)	0.92 (0.87–0.94)
	MSPSS Friends		0.92 (0.86–0.95)	0.92 (0.87–0.96)
	MSPSS Significant Others		0.89 (0.81–0.94)	0.89 (0.82–0.95)

^a^Weighted kappa coefficient.

^b^Intraclass correlation coefficient.

^c^Lin’s concordance correlation coefficient.

## Discussion

The aim of this study was to translate, adapt and psychometrically evaluate the MSPSS for use in a Swedish context. In the present sample, the findings demonstrated that MSPSS has good psychometric properties with regard to factor structure, construct validity (i.e. known group validity), internal consistency and reproducibility.

In order to reach a psychometric sound Swedish version of the MSPSS we conducted the translation according to WHO:s official process of translation. This implies a “conceptual equivalence rather than literal translation” The strength of this approach has been discussed by Maneesriwongul & Dixon [42]. The cultural adaption has been carried out with the help of a bilingual person familiar with both cultures. The Swedish agreed-upon version differed slightly for the items belonging to the dimension Significant others, in which we used “an important person in my life close by” rather than “a special person”. This difference was discussed with Professor Zimet, the constructor of the MSPSS, who claimed that even if there was a slight difference between the translated version and the original version, the items still reflect the underlying construct. Also the psychometric evaluation in the present study supports this conclusion as this dimension was reproduced by the factor analysis.

All items and scale scores were negatively skewed distributed, with a ceiling effect. Problems with social desirability cannot be ignored, but the large number of women living with a partner and/or having children indicates that the group in general had good social support. Skewed distributed data may have a problem with poor discrimination, i.e. reduced sensitivity and responsiveness. However, reduced discrimination is probably a minor problem for the items in the MSPSS as all response categories were used [34]. Related to the endorsement of the items, the level of missing data was low and only related to a few of the participants. This indicates that the items are easy to understand and relevant for respondents taking the test.

We used an explorative approach to confirm the original factor structure of the MSPSS. The hypothesis was that the three-factor solution, that has been suggested by the constructor [9,17] and others who have validated the English version of the MSPSS [23], should be reproduced using the Swedish version. Although confirmatory factor analyses (CFA) are more appropriate and common when a hypothesized measurement model should be evaluated, exploratory approaches can be used for the same purpose [43]. The reason was based on the sample size that was somewhat limited for CFA. According to the general rule of thumb, the sample size should be at least 10–15 individuals per item for an exploratory factor analysis or principal component analysis [32]. From this perspective, our sample size was large enough for the factor analyses on the main and reference sample. As hypothesized, the original three-factor model was reproduced on the main sample of women with hirsutism. The same factor structure was also demonstrated in the cross-validation on the reference sample of nursing students. These findings support the use of the subscale scores but not the use of a total score. Although the correlations were strong between all factors, a higher-order factor analysis is needed before stronger conclusions can be drawn.

The extraction of factors has, for a long time, been criticized as the most critical part when conducting exploratory factor analysis or principal component analysis [44]. To reach a more objective decision, we used the Kaiser criteria with eigenvalues >1 to extract the number of factors and then confirmed these using parallel analysis. The replication of the three-factor structure, strong factor loadings, absence of double loadings (i.e. items with strong factor loadings on two or more factors), largely explained variance and small uniqueness values (1-communality) in both the main sample and in the cross-validation which indicates that the Swedish version of the MSPSS has an equivalent factor structure as the original English version. However, it should be emphasized that factor analyses are sample dependent methods and the findings are therefore difficult to generalize [32]. Related to this, one limitation with the present study was that both samples were homogenous regarding gender and age. Before a stronger conclusion can be drawn, the Swedish version needs to be psychometrically evaluated in other groups, in particular men and older persons.

Known group validity, an aspect of construct validity, was supported as the MSPSS had the ability to discriminate between women with hirsutism and nursing students. Women with hirsutism scored significantly lower social support compared to nursing students, as was hypothesized according to an earlier study [36,37]. According to the Cohen’s d effect size, there was a greater difference for the subscales Friends and Significant others compared to Family. One reason for that could be that sick people, even the youngest, seem to perceive family as a more stable source of support compared to friends, which has been noted in earlier studies [45,46]. Known-group validity is not only a test of construct validity, if a scale successfully discriminates between groups, it also supports sensitivity and responsiveness [34].

Internal consistency was good for all the MSPSS scales, both in the main and reference sample. There is always a risk that too high alpha values reflect redundant items (i.e. items that measure the same aspect of the construct). However, there is no clear definition of what a too high alpha value is. Streiner and Norman [47] suggest that alpha values should not exceed 0.9 while Nunnally and Bernstein [30] state that clinical scales should have alpha values of at least 0.9. High alpha values have also been seen in other studies using MSPSS, but the level seems to vary between diagnose groups and ethnicity [17,23,48]. One often recommended solution to this problem is to shorten the scale [49]. This probably works well for scales with a large item pool, but is less appropriate for scales with fewer items, such as the MSPSS. Each subscale in the MSPSS includes only four items and reducing these should probably have a serious impact on other aspects, such as content validity. In addition, although two inter-item correlations were very strong (>0.9), the rest were in the range of 0.42 and 0.87. For the majority of the items in the MSPSS, redundancy seems to be a minor problem.

The reliability in terms of reproducibility was satisfactory for both items and scales. All except item 5 (“I have a special person who is a real source of comfort for me”) demonstrated good or very good reproducibility. One explanation for this may be related to the Swedish translation and adapting of “Significant others” into “an important person in my life close by”. It is possible that this adaption makes it difficult to distinguish items from the subscale Significant others and Friends. However, these tests were conducted on the reference sample and for that, there was only a moderate correlation between these two subscales. In addition, the rest of the items in the subscale Significant others (items 1, 2 & 10) demonstrated good reproducibility. The reproducibility for all of the MSPSS scales was very good, assessed with both intraclass correlations and Lin’s concordance correlation coefficient. Despite the fact that the sample size for these analyses was somewhat limited, the small confidence intervals indicate that the uncertainty was small and that the same level of reproducibility therefore can be expected in other samples as well.

Further research is warranted to confirm the factor structure with confirmatory factor analysis. This should be done in a larger sample including more men and older persons. It seems also important to determine the predictive validity in terms of cutoff levels and to evaluate differential item functioning for groups often compared, such as gender, age and ethnicity.

## Conclusions

### The Swedish version of the MSPSS offers a simple way to measure perceived social support

In the present study sample, dominated by younger women, the MSPSS presented sound psychometric properties as shown in previous validation studies on the original English version. The scale is multidimensional, including three unique dimensions. Equivalent factor structure between the Swedish and the original English version facilitates international comparisons. The short format makes it into a useful tool, especially for large surveys and in clinical praxis.

## Competing interests

The authors have no competing interests.

## Authors’ contributions

MPE, contributions with design, collection of data, interpretation of data, intellectual content, and drafting the manuscript. EB, contributions with design, interpretation of data, intellectual content, and drafting the manuscript. ML, contributions with design, interpretation of data, intellectual content, and drafting the manuscript. KÅ, RN, contributions with design, collection of data, data analysis, interpretation of data, intellectual content, and drafting the manuscript. All authors read and approve the final manuscript.
